# Hamartochondroma Pleural Lesion Mimicking Liposarcoma: A Case Report

**DOI:** 10.3390/curroncol29050281

**Published:** 2022-05-11

**Authors:** Gabrielle Drevet, Erik Kovacs, Lara Chalabreysse, Delphine Gamondes, François Tronc

**Affiliations:** 1 Department of Thoracic Surgery and Lung Transplantation, Louis Pradel Hospital, Hospices Civils de Lyon, 69677 Bron, France; erik.kovacs@intradef.gouv.fr (E.K.); francois.tronc@chu-lyon.fr (F.T.); 2 Department of Pathology, Louis Pradel Hospital, Hospices Civils de Lyon, 69677 Bron, France; lara.chalabreysse@chu-lyon.fr; 3 Department of Radiology, Louis Pradel Hospital, Hospices Civils de Lyon, 69677 Bron, France; delphine.gamondes@chu-lyon.fr

**Keywords:** benign tumor, pleural tumor-like lesion, hamartochondroma

## Abstract

Heterogeneous masses developing in the pleural cavity are most often malignant and can pose diagnostic challenges. Fibrous tumors of the pleura, liposarcoma, thymoma or lipoma most frequently affect this anatomic area. Surgical exploration and resection are often mandatory to make the definitive diagnosis. We report the case of a 54-year-old women who presented with an epigastric and right sub costal pain. A complete preoperative workup revealed a large tissular and fatty mass in the right costo-diaphragmatic angle suggestive of liposarcoma. Surgical resection resulted in the surprising diagnosis of hamartochondroma.

## 1. Introduction

Pleural masses or effusions are a common clinical presentation. The challenge for the clinician is to make a precise diagnosis. Although these lesions may share some similarities, clinical history, imaging techniques and sometimes biopsy can help distinguish a benign pleural lesion from a malignant one. In rarer cases, there may still be some doubt. Herein, we report the case of a patient presenting with a heterogeneous pleural mass suggestive of liposarcoma.

## 2. Case Presentation

**Figure 1:**
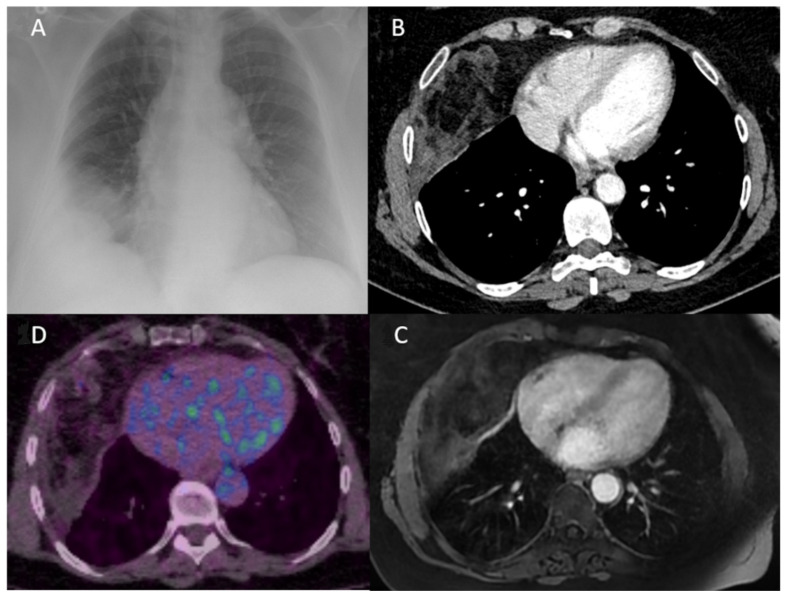
(**A**) Chest X-ray showing a non-systematized opacity of the right costo-diaphragmatic angle making the diagnosis between pleural effusion and thoracic mass difficult. (**B**) CT scan of the chest (axial view) confirming the presence of a heterogeneous intra thoracic mass not appearing to be in the lung parenchyma. (**C**) An axial T1-weighted MR image of the chest identifying a tumorous chest wall mass with both fat and tissue components, suggestive of liposarcoma. (**D**) 18-fluorodeoxyglucose (FDG) positron emission tomography showing a low hypermetabolism of this lesion with a standardized uptake value of 1.8.

A 54-year-old woman with a medical history of severe obesity, myocardial infarction, hypertension and resection of uterine fibroids presented with an epigastric and right sub costal pain that had been progressing for 1 year. Physical examination did not reveal much information except a pain on peri-umbilical palpation. She had no nicotine or alcohol-dependent behavior. Vital signs were within normal values. Laboratory tests did not show any infectious syndrome. The electrocardiogram did not reveal any anomaly. The abdominal ultrasound revealed gallstones and a chest X-ray, routinely performed, showed an opacity in the right costo-phrenic angle (Figure 1A). Thus, a complement of investigation was performed with a thoraco-abdominopelvic CT scan. The CT scan showed a heterogeneous pleural mass, with a fatty and tissue component, without calcification and not appearing to invade neighboring structures or the chest wall (Figure 1B). Chest MRI was then performed to better understand the nature of this lesion. The MRI confirmed a heterogeneous mass with both fatty and tissue components of antero-inferior right subpleural location, without arguments for adjacent organ or chest wall invasion, but whose appearance remains suggestive of a liposarcoma (Figure 1C). An 18-fluorodeoxyglucose positron-emission tomography scan showed a low hypermetabolism of the lesion with a standardized uptake value of 1.8 (Figure 1D). No other FDG uptake was observed.

**Figure 2:**
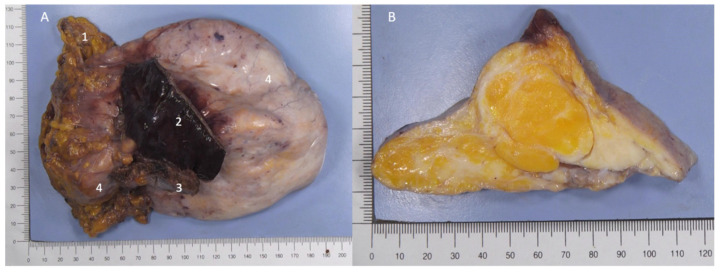
(**A**) Hamartochondroma consisting of a firm, bumpy-contoured, encapsulated mass measuring 16.5 × 11 × 5.8 cm and weighing 442g. 1: pericardial fat. 2: lung parenchyma. 3: diaphragm. 4. hamartochondroma. (**B**) After fixation, a yellowish and whitish tumor is observed. There is contact with the right lung, diaphragm and pericardium without invasion.

**Figure 3:**
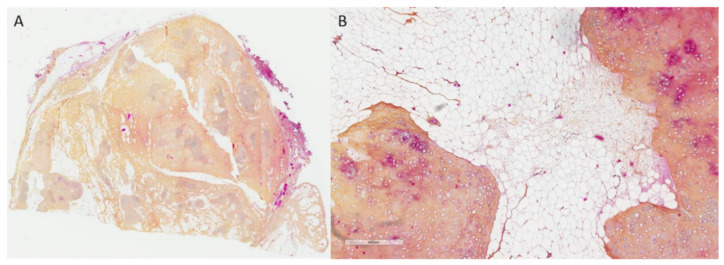
(**A**). Original × 25. At low magnification, lobules of cartilaginous tissue intermingled by fibrovascular and adipose tissue. (**B**). Original × 100. Lobules of mature cartilage with cytologically bland cells and deep clefts lined by bronchiolar type epithelium.

In front of this unusual pleural mass, an ultrasound-guided biopsy was performed, which confirmed the presence of a heterogeneous tissue mass, not invading the chest wall, and made the diagnosis of hamartochondroma. The patient’s medical chart has been presented to the multidisciplinary tumor board. Our difficulty was that, on the one hand, the appearance on the MRI was very suggestive to our radiologists of a liposarcoma, and on the other hand, the absence of fixation on the PET scan was rather in favor of a less aggressive lesion. As some subtypes of liposarcoma can present with a low FDG uptake [1], a surgical resection was decided in this young patient with an operable tumor, allowing a precise diagnosis and its treatment at the same time. During surgical resection, only a few inflammatory adhesions without direct invasion with the lung and diaphragm were noted. The mass seemed to develop within the pericardial fat. No chest wall or pericardial resection was required. The final pathology of the surgical specimen confirmed the complete resection of a hamartochondroma (Figure 2). Although the radiological and macroscopic appearance was in favor of an aggressive lesion, the microscopic appearance was rather typical of a hamartochondroma. On histological examination, it was a mixed adipose-fibrous and cartilaginous tissue in islands. Some congestive vessels, mature adipose and cartilaginous tissue were observed. The pulmonary parenchyma in contact was very congestive. No atypia, mitosis and lipoblasts or necrosis were noted (Figure 3). There was no overexpression of the anti-MDM2 antibody. There was no evidence of malignancy. The post-operative course was uneventful, and the patient was discharged eight days after surgery. The patient is free of recurrence 3 months post operatively. Epigastric and right sub costal pain recurred a few weeks after the intervention and were finally imputed to a pancreatitis secondary to a pancreas divisum.

## 3. Discussion

Most often, individualized and heterogeneous masses of the pleural cavity are malignant, with the most common diagnosis being malignant fibrous tumor of the pleura, liposarcoma, thymoma and pleural metastasis. Sometimes, benign tumors can also be found, such as localized fibrous tumor of the pleura or pleural lipoma [2]. Hamartochondroma developed in the pleural cavity has never been described before. This lesion had an atypical anatomic location and radiological presentation. The typical radiological presentation of a hamartochondroma is a round homogeneous opacity in the periphery of the lung with calcifications in 10% of cases [3] and for which a fatty component is identified in 50% of cases [4]. The majority of these tumors are very slow growing and usually diagnosed at a small size (between 4 and 90 mm), but a few cases of giant pulmonary hamartoma in adults have been reported [5]. No giant mediastinal or pleural hamartoma has been reported so far. Liposarcomas, on the other hand, have various radiological presentations based on their histological subtype. Well-differentiated liposarcoma may appear as a large mass, predominantly fatty with the presence of internal septations and nodular areas of non-adipose tissue. The extent of fat within a liposarcoma decreases as the tumor becomes more aggressive and less differentiated [6]. In our case, the CT scan and MRI showed a large heterogeneous mass with some areas of fat and soft tissue leading to the misdiagnosis of liposarcoma. On a large suspicious mass, diagnosis is not based only on imaging findings. Fine needle or thoracoscopic biopsy is performed to establish the diagnosis and to decide whether the patient should undergo neoadjuvant chemotherapy [7]. An ultrasound-guided biopsy was performed on our patient, but because the diagnosis of hamartochondroma was not consistent with what could be seen on the imaging, a surgical resection was decided.

Hamartochondromas are benign tumors derived from peribronchial mesenchymal tissue and usually arise from the lung parenchyma (accounting approximatively for 8% of pulmonary neoplasm) or from the bronchi (accounting for 1–20% of all pulmonary hamartomas) [3,8]. One case of hamartoma developed at the expense of the visceral pleura, and growing outside the lung has been reported [9]. Rare cases have been described in the mediastinum. A case of hamartochondroma arose from the peribronchial tissue in the scissure and fused to the mediastinum [10]. Other cases of hamartoma have been described in the posterior mediastinum initially leading to the misdiagnosis of mycotic aortic aneurysm, bronchogenic cyst or neurogenic tumors [11,12,13,14]. These tumors were well encapsulated and independent of the lung and the bronchial tree. One of them was multifocal. In the case we describe, it is difficult to determine the exact origin of the lesion. The lesion appeared to be “free” in the pleural cavity. There were adhesions with the lung and the diaphragm but without direct invasion. The pericardial fat resected at the contact was the site of scattered lymphoid structures within an adipose-fibrous tissue, suggesting an origin at the expense of this structure, without being able to conclude with certainty.

For all these tumors, the diagnosis was made on a surgical specimen. Surgical resection should be performed in symptomatic patients or in patients presenting with a solitary mass or nodule, which cannot be differentiated from malignancy [15,16], which was our case. Despite the benign nature of this lesion and a complete resection, the risk of recurrence exists [15]. Furthermore, a relationship between hamartoma and lung cancer has been highlighted. The risk for lung cancer in pulmonary hamartoma patients was estimated to be about 6 times as high as the age-, sex- and ethnicity-adjusted rate expected for the general population [17,18]. Consequently, a regular follow-up in this kind of patient would not be illogical.

## 4. Conclusions

Although it is a rare development in the pleural cavity, the diagnostic of hamartochondroma should be considered in the differential diagnosis of a heterogeneous mass in that anatomic area.

## Data Availability

All data generated or analyzed during this study are included in this article. Further enquiries can be directed to the corresponding author.
